# Testing the assumptions of the pyrodiversity begets biodiversity hypothesis for termites in semi-arid Australia

**DOI:** 10.1098/rsos.172055

**Published:** 2018-04-25

**Authors:** Hayley Davis, Euan G. Ritchie, Sarah Avitabile, Tim Doherty, Dale G. Nimmo

**Affiliations:** 1 School of Life and Environmental Sciences, Centre for Integrative Ecology (Burwood campus), Deakin University, Geelong, Victoria 3220, Australia; 2 Department of Zoology, La Trobe University, Bundoora, Victoria 3086, Australia; 3 School of Environmental Science, Institute for Land, Water and Society, Charles Sturt University, Albury, New South Wales 2640, Australia

**Keywords:** fire ecology, mallee, pyrodiversity, landscape heterogeneity, fire management, invertebrates

## Abstract

Fire shapes the composition and functioning of ecosystems globally. In many regions, fire is actively managed to create diverse patch mosaics of fire-ages under the assumption that a diversity of post-fire-age classes will provide a greater variety of habitats, thereby enabling species with differing habitat requirements to coexist, and enhancing species diversity (the pyrodiversity begets biodiversity hypothesis). However, studies provide mixed support for this hypothesis. Here, using termite communities in a semi-arid region of southeast Australia, we test four key assumptions of the pyrodiversity begets biodiversity hypothesis (i) that fire shapes vegetation structure over sufficient time frames to influence species' occurrence, (ii) that animal species are linked to resources that are themselves shaped by fire and that peak at different times since fire, (iii) that species’ probability of occurrence or abundance peaks at varying times since fire and (iv) that providing a diversity of fire-ages increases species diversity at the landscape scale. Termite species and habitat elements were sampled in 100 sites across a range of fire-ages, nested within 20 landscapes chosen to represent a gradient of low to high pyrodiversity. We used regression modelling to explore relationships between termites, habitat and fire. Fire affected two habitat elements (coarse woody debris and the cover of woody vegetation) that were associated with the probability of occurrence of three termite species and overall species richness, thus supporting the first two assumptions of the pyrodiversity hypothesis. However, this did not result in those species or species richness being affected by fire history *per se.* Consequently, landscapes with a low diversity of fire histories had similar numbers of termite species as landscapes with high pyrodiversity. Our work suggests that encouraging a diversity of fire-ages for enhancing termite species richness in this study region is not necessary.

## 1. Introduction

Fire shapes the structure and function of ecosystems around the world and has done for millennia [1]. Recent and projected increases in wildfire mean that fire management is a chief concern of conservation biologists and land managers in many regions across the globe [2,3]. A challenge to land managers in fire-prone regions is to provide for multiple species that may have varying responses to fire [4]. To meet this challenge, land managers often impose fire on landscapes to provide a diverse mosaic of vegetation patches that differ in their fire history (patch mosaic burning), thereby increasing ‘pyrodiversity’ (i.e. the diversity of fire histories) [4]. It is hoped that such burning will provide a broader array of niches such that ‘pyrodiversity begets biodiversity’ (referred to hereafter as the ‘pyrodiversity hypothesis') [4,5]. Despite its popularity, studies of the relationship between pyrodiversity and biodiversity have reported mixed results—some showing a positive relationship [6–10] and others no clear relationship [11–16].

**Figure 1.**
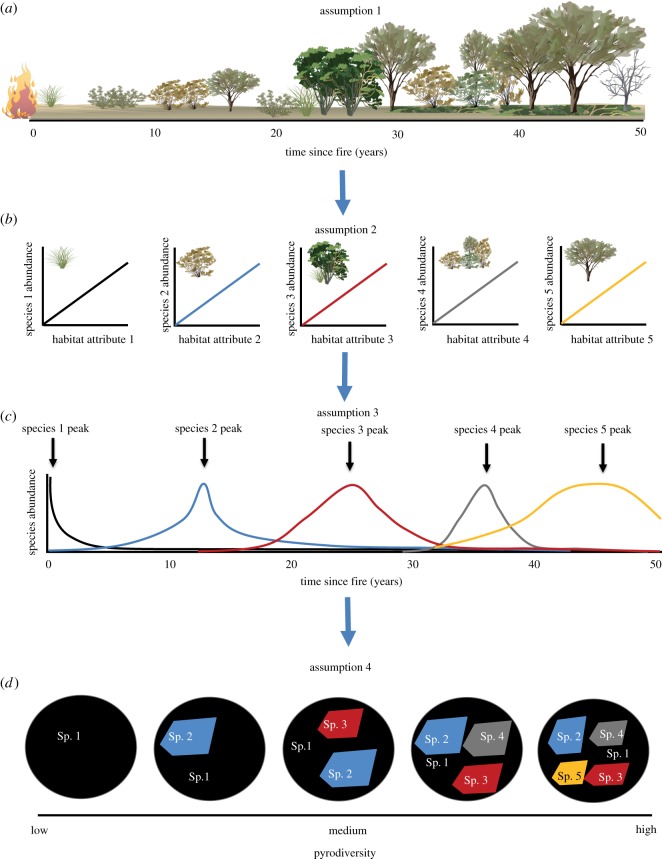
Illustration of the assumptions underlying the pyrodiversity begets biodiversity hypothesis. (*a*) The first assumption is that fire is a strong driver of vegetation succession such that vegetation changes with time-since-fire. (*b*) The second assumption is that animal species are tightly related to particular habitat features that change along the time-since-fire continuum. (*c*) The third assumption is that the strong relationship between animal species and fire-affected habitat features results in species displaying relationships with time-since-fire that vary based on the changes in their preferred habitat feature, peaking in their abundance or probability of occurrence when the habitat feature is most abundant. (*d*) A series of fire mosaics that differ in their ‘pyrodiversity’, from landscapes composed of a single fire-age to landscape composed of five fire-ages. Different colours represent different fire-age classes that are suitable for different species: black = 0–5 years post-fire, blue = 10–15 years post-fire, red = 20–30 years post-fire, grey = 30–40 years post-fire, yellow = 40–50 years post-fire.

Although ‘pyrodiversity’ encapsulates a number of concepts regarding how the spatio-temporal properties of fire influence biodiversity [17], one common interpretation is that landscapes high in pyrodiversity have a greater diversity of fire-ages, whereas landscapes low in pyrodiversity have a more uniform fire history [12,18]. Biodiversity will be higher across more pyrodiverse landscapes due to greater landscape heterogeneity providing habitat for a broader array of species. In order for this version of the pyrodiversity hypothesis to be supported, a series of assumptions must be met (figure 1). First, fire must exert a strong influence on habitat structure by setting in train vegetation successional dynamics that play out over time (assumption 1, figure 1*a*) [19]. Second, species niches must be dependent on the availability of resources that change along the time-since-fire axis. Time-since-fire would, therefore, be expected to affect species occurrence indirectly through the provision of varying post-fire successional stages of habitat (assumption 2, figure 1*b*) [20]. Third, within an animal community, individual species or groups of species must be reliant on resources that peak at different stages during post-fire succession [21]. This would result in different animal species peaking at different times since fire (assumption 3, figure 1*c*), in some instances including multiple peaks [3]. Finally, providing each of these assumptions are met, increased diversity of fire-ages may provide for a broader array of species, resulting in increased landscape-scale animal diversity (assumption 4, figure 1*d*).

Termites (Blattodea) are functionally critical to many fire-prone ecosystems around the world [22,23]. They perform vital ecosystem functions including decomposition, nutrient cycling [24] and soil maintenance [25]. Termites also are a critical food resource [26,27] and create habitat for other species [28]. Studies of the effects of pyrodiversity on termites have questioned the applicability of the pyrodiversity hypothesis for this group. For instance, Davies *et al*. [15] found that termite communities were not more diverse in plots subject to a more diverse fire history (i.e. variability in frequency and season) in savannah ecosystems of South Africa, while Avitabile *et al*. [29]—working in semi-arid mallee vegetation to the north of the current study region—showed that more diverse fire mosaics did not have more diverse termite assemblages. In both cases, the termite communities were largely resistant to measures of fire history (thus violating assumption 3 of the pyrodiversity hypothesis). Similar patterns have been observed for ants [14,30], leading Bowman *et al*. [31] to argue that ‘these species-rich communal organisms are possibly better buffered against changes in fire regimes than vertebrates’ [31].

Here, we aim to test the assumptions of the pyrodiversity hypothesis using termite communities from semi-arid Australia. We employed a hierarchical experimental design that included 100 sites nested within 20 study landscapes—each 1256 ha in size—carefully chosen to represent a gradient in pyrodiversity (i.e. from landscapes with a single fire-age to those with several fire-ages). We collected data on both vegetation structure and termite occurrences at each of the 100 sites, quantified the properties of the 20 landscapes within which those sites were nested (including pyrodiversity), and related landscape-scale pyrodiversity to the diversity of the termite community. This multi-scaled design allowed us to test each of the four assumptions of the pyrodiversity hypothesis, permitting insight into the mechanisms underpinning either support or rejection of the hypothesis.

Our specific study questions—based on the four assumptions of the pyrodiversity hypothesis—were as follows:

(1) Does fire history affect termite habitat resources?
(2) Does vegetation structure influence termite occurrence?
(3) Does fire history affect termite occurrence?
(4) Does the diversity of fire history within a landscape positively affect termite species richness?

## 2. Methods

### 2.1. Study area

**Figure 2.**
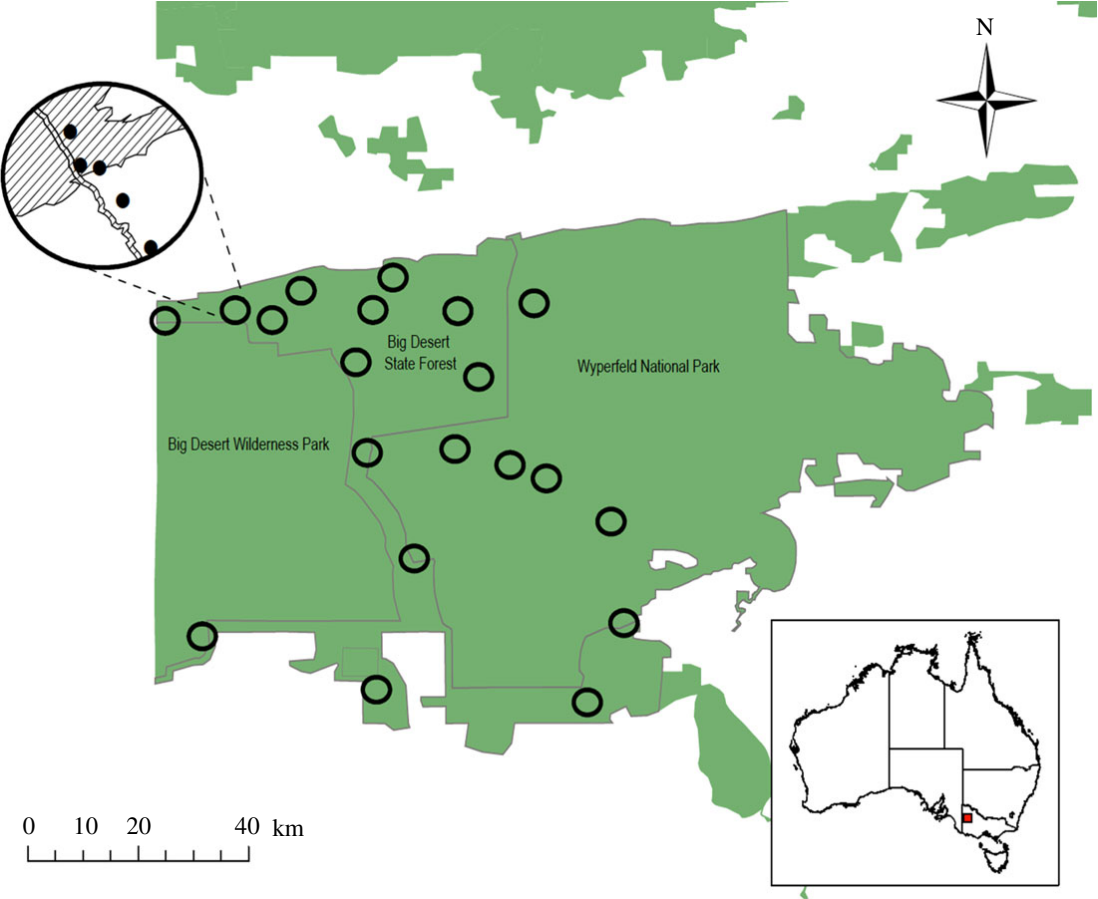
The Big Desert study region in northeastern Victoria, Australia. Open black circles represent the 20 study landscapes positioned across the region. Five sampling sites were clustered within each of the 20 study landscapes (*n *= 100 sites), represented by solid black circles in the magnified landscape. The hatched and white areas in the magnified landscape represent the different fire-age classes present.

The approximately 7000 km^2^ study area is located in northwestern Victoria, Australia, and encompasses the Big Desert Wilderness Park, Big Desert State Forest and Wyperfeld National Park (hereafter ‘Big Desert’; figure 2). This region lies south of the study region of a large project on the effects of pyrodiversity on biodiversity, the Mallee Fire and Biodiversity Project, which also included a study on the impacts of fire on termites [29]. The region experiences a semi-arid climate, with hot, dry summers (mean maximum temperature = 30.9°C), cooler winters (mean maximum temperature = 14.5°C) and mean annual rainfall of 327.1 mm (Australian Bureau of Meteorology 2014). The topography of the area comprises irregular, east–west orientated dune fields interspersed with broad sand plains and relict sandstone ridgelines [32]. Three ecological vegetation classes dominate the study region: dunefield heathland, heathy mallee and sandstone ridge shrubland. Dunefield heathland occurs on deep sands and is characterized by low growing (less than 2 m) heathy vegetation, generally without trees. Heathy mallee consists of a ‘mallee’ eucalypt canopy, which is a multi-stemmed form of *Eucalyptus* arising from an underground lignotuber. Heathy mallee has a diverse understorey of heathy shrubs and *Triodia scariosa*, occurring primarily on infertile dunes and plains. Sandstone ridge shrubland occurs on the sandstone ridgelines and is dominated by *Melaleuca uncinata*, sometimes co-dominant with mallee eucalypts [32].

Semi-arid shrublands and mallee ecosystems of southern Australia experience large wildfires (greater than 10 000 ha) approximately every 35 years [33]. However, fires are more frequent in the Big Desert because the higher moisture availability allows the development of large, continuous fuel loads [34]. This results in large fires occurring as frequently as every 5–20 years [32]. Fires include wildfire and prescribed burning, with the latter used to reduce fuel loads for asset protection and biodiversity conservation [33]. The high continuity of fuel and short vegetation height means that fires are almost always stand-replacing, with burned areas set back to ‘year-zero’ in a successional sense. As such, post-fire-age classes—‘time-since-fire’—can be assigned to an area based on when it last burned. Dominant vegetation—most notably mallee *Eucalyptus*—has the capacity to regenerate from underground lignotubers following fire by coppicing new stems.

### 2.2. Experimental design

We used a natural experimental design and space-for-time substitution to sample biodiversity in sites that have experienced differing fire histories. Sampling occurred within 20 study landscapes. Each landscape was a circular area 4 km in diameter (12.65 km^2^; following Taylor *et al*. [12]). Landscapes were stratified to represent variation in: (i) the spatial extent of fire-age classes: recently burned (less than 11 years post-fire), mid-successional (11–35 years) or long unburned (greater than 35 years) vegetation and (ii) the number of fire-age classes within a landscape. Study landscapes were positioned greater than 2 km from neighbouring landscapes to enhance independence (figure 2). The fire history of study landscapes was determined from maps spanning 1958–2014, which were constructed using a combination of satellite imagery and expert local knowledge (accessed January 2014; https://www.data.vic.gov.au/data/dataset/fire-history-overlay-of-most-recent-fires).

Each landscape contained five sampling sites (20 study landscapes × 5 sites, *n* = 100 sites in total) that were selected using ArcGIS (ESRI 2011). Sites were positioned based on the proportional spatial extent of a given fire-age class within a study landscape using area-proportionate sampling (following [12]) according to the following criteria: extent of fire-age class ≤ 20% = 1 site; 21–40% = 2 sites; 41–60% = 3 sites; 61–80% = 4 sites; 81–100% = 5 sites. Each site consisted of a 50 m transect. Sites were located approximately 50 m from vehicle tracks to enable access and situated at least 200 m from neighbouring sites to increase sampling independence between sites.

### 2.3. Data collection

#### 2.3.1. Termite baits

Buried cellulose baits (toilet paper rolls) were used to sample termites at each of the 100 sites in 2014. Cellulose baits are an effective technique to sample termite species presence and diversity [35,36], and are particularly effective within semi-arid environments [29,36]. One grid of six toilet paper rolls (unbleached, unscented, 400 sheet, 2-ply) was established at each site (*n *= 600 toilet paper rolls) in the centre of a 50 m transect. Rolls were spaced 2 m apart in 2 × 3 grid, following [27]. Rolls were buried upright just below (approx. 3 cm) the soil surface, with a length of coloured flagging tape tied through the centre to stop the roll unravelling and to aid relocation in the field [29]. Grids were installed during mid-April, and left *in situ* for three months to allow for termite colonization [35,36]. Toilet rolls were carefully excavated and visually assessed to measure termite-caused decomposition based on the presence of termites, termite foraging galleries and hollowed areas on the rolls indicative of feeding activity. Individual termites were collected from the baits, with an emphasis on collecting soldier castes, which are members of the colony with distinct head or mandible morphology that allows species identification when viewed under a microscope. Collected specimens were stored in 70% ethanol until examination in the laboratory.

#### 2.3.2. Active searches

To supplement the termite baiting, we carried out active searches for termite species at each site (*n *= 100 belt transects) (following [37,38]). Woody debris was surveyed for termites within a 10 m × 50 m belt transect (5 m either side of the 50 m transect line). Time-constrained surveys were conducted for a maximum of 40 min, or until all of the woody microhabitats within the belt transect had been examined. This involved searching under surface woody debris—such as fallen limbs, logs and stumps—examining attached dead mallee stems, and digging up protruding remnants of mallee roots. Woody debris was pulled apart to expose termite colonies, and soldier castes were collected from each colony and stored in 70% ethanol. Active searches were conducted once during the study period, over July and August, at the same time as the bait inspection. Specimens collected from the active searches and the baits were preserved separately to enable comparison of species detections from the two survey methods.

#### 2.3.3. Species identification

Specimens were examined under a high-powered dissecting microscope (Nikon SMZ1000), and were identified to species level, when possible, using a regionally appropriate reference collection (gathered during the Mallee Fire and Biodiversity Project [27]) and identification keys [39]. When specimens could not reliably be identified to species level, but were clearly separate species based on distinguishable differences in head morphology, they were identified to genus level using keys and given a unique species code.

#### 2.3.4. Habitat sampling

Vegetation structure was surveyed along the 50 m transect, following the methods outlined in Haslem *et al*. [19]. A 2 m structure pole was placed at metre intervals (starting at metre 1) along the transect (*n *= 50 points per site). At each interval, ground cover was categorized as one of plant matter, leaf litter, cryptogamic crust or bare ground. To characterize vegetation structure at various heights, the number of vertical contacts of vegetation on the pole was recorded at four different height intervals (less than or equal to 0.5 m, 0.5–1 m, 1–2 m, greater than 2 m). At each height interval, contacts were separated as belonging to one of several life forms: grass, *T. scariosa* (hereafter Triodia), herb, eucalypt shrub (any *Eucalyptus* species less than 3 m in height), shrub (any woody, non-*Eucalyptus* species less than 3 m in height), tree (any tree species greater than 3 m in height) or dead matter. The differentiation between eucalypt trees and shrubs is relevant to termites because the woody resources associated with small versus tall eucalypts differ. For instance, larger mallee trees are more likely to retain high covers of defoliating bark, hollows and dead limbs.

A major habitat component likely to influence termite presence and diversity is the amount of woody debris [40]. Termites use woody debris for food and shelter [41,42]. Therefore, the volume (cubic centimetres) of surface coarse (greater than 2.5 cm diameter) woody debris present at sites was assessed throughout the belt transect. Volume (*V*) was calculated for each piece of woody debris by first measuring the length (*L*) and radius of each piece (*r*) (centimetres), then using the following equation to estimate a volume in cubic centimetres for each piece of woody debris:

$$V=L\times \pi \times r^{2}.$$

These values for each piece of woody debris at a site were then added together to attain the estimated volume of coarse woody debris at each site. All measurement estimations were conducted by the same individual to avoid sampling inconsistencies.

### 2.4. Statistical analysis

#### 2.4.1. Does fire affect termite habitat resources?

Generalized additive mixed models (GAMMs) were used to analyse how fire affects the availability of termite habitat resources following Haslem *et al.* [19]. Habitat response variables (outlined in table 1) were modelled as a function of two fixed effects: (i) a continuous variable indicating the number of years since a site last experienced fire (time-since-fire) and (ii) a categorical variable indicating the broad vegetation type a site was located within (i.e. dunefield heath, heathy mallee or sandstone ridge shrubland). An interaction term between time-since-fire and vegetation type was included which allowed a separate relationship (or ‘smoothed term’) between the response variable and time-since-fire to be generated within each of the vegetation types [43]. ‘Landscape’ was fitted as a random factor in the models to allow for possible spatial autocorrelation in the data due to the sites being clustered within landscapes [44].

**Table 1.** Variables used in models assessing how habitat resources respond to time-since-fire, how termites are affected by habitat resources and how termite species respond to fire in the Big Desert.

spatial scale	variable	description
site scale	time-since-fire (years)	number of years since fire has occurred at a site
	vegetation type	categorical variable describing the broad vegetation type of a site (heathy mallee, dunefield heath, or sandstone ridge) based on ecological vegetation classes
	coarse woody debris (cm^3^)^a^	the volume (cm^3^) of surface lying coarse (greater than 2.5 cm diameter) woody debris. See text for further detail
	woody cover (%)^a^	the proportional cover of woody vegetation during 50 m line transect at each site, measured as the number of hits of woody cover/the total number of possible hits along the 50 m transect
	leaf litter (%)^a^	the proportion of hits of leaf litter along a 50 m transect at each site, measured as the number of hits of leaf litter/the total number of possible hits along the 50 m transect
	habitat complexity (%)^a^	the proportion of hits of any vegetation during 50 m line transect at each site, measured as the number of hits of any vegetation attribute/the total number of possible hits along the 50 m transect
	Triodia cover^a^	the proportion of hits of spinifex during 50 m line transect at each site, measured as the number of hits /the total number of possible hits along the 50 m transect
	topography	binary variable describing the land form of the site: either dune (including dune crest, dune slope) or a swale
landscape scale	fire diversity	Shannon–Wiener diversity index of extent of fire-age classes present in the landscape
	recently burned (%)	proportional cover of areas <11 years since fire within each study landscape
	mid-successional (%)	proportional cover of areas 11–35 years since fire within each study landscape
	long unburned (%)	proportional cover of areas >35 years since fire within each study landscape
	vegetation type (%)	proportional cover of ecological vegetation classes within each study landscape. Either dunefield heath, heathy mallee or sandstone ridge shrubland

^a^Variables that were response variables when modelling how they respond to time-since-fire, and predictor variables when they were used to model termite species’ occurrence and richness.

#### 2.4.2. Does vegetation structure influence termite occurrence?

To examine the effects of habitat resources on termite species occurrence and richness, we used generalized linear mixed models (GLMMs). Species occurrence was analysed based on species' presence or absence at sites. The presence or absence of individual species across the sites was assumed to follow a binomial distribution with a logit-link function [44]. Species richness was specified as following a Poisson distribution and a log link [44]. Predictor variables were included as fixed effects and were both continuous and categorical (table 1). Only variables with Pearson's correlation coefficients less than 0.6 were included within the same model [45]. To allow the direct comparison of variable coefficients, the continuous predictor variables were standardized (mean = 0, s.d. = 1).

For each response variable, a series of candidate models were generated that included all possible combinations of the six predictor variables, as well as a null model that included only the intercept (and ‘landscape’ as a random factor). Models were ranked according to Akaike's information criterion corrected for small sample sizes (AIC_c_) [46] and the models deemed most parsimonious were those with the lowest AIC_c_ values. The difference (Δ*_i_*) between the best supported and lower-ranked models was calculated as a comparison of the level of support for each model [47]. Models with Δ*_i_* < 2 were considered to have substantial support [47]. Akaike weights (*w_i_*) were calculated to evaluate the relative strength of candidate models, with higher *w_i_* indicating models more likely to explain the data [48]. Models with *w_i_* > 0.9 were considered to be clearly the best fit for the data [47]. When no single model was identified as clearly being the best (*w_i_* > 0.9), model averaging was undertaken to evaluate the influence of variables by assessing their coefficient estimates. Predictor variables were considered to be important when the 85% confidence intervals for the averaged coefficient estimates did not overlap zero [49].

#### 2.4.3. Does fire affect termite occurrence?

GAMMs were built to examine the effects of fire and vegetation type on termites at sites. Response variables were again species presence/absence, assuming a binomial distribution and logit-link function, and total species richness assuming a Poisson distribution with a log link function. The structure of these models was similar to those outlined in the previous GAMMs of habitat resources, with an interaction term fitted between the predictor variables to examine differences in responses between vegetation types and ‘landscape’ fitted as a random factor.

#### 2.4.4. Does the diversity of fire history within a landscape positively affect termite species richness?

While the question of whether pyrodiversity begets biodiversity is an assemblage level question, it is also important to model the response of individual species to fire mosaic properties as other studies have shown individual species to be strongly related to the spatial extent of fire-ages or vegetation types [18,50]. We used generalized linear models (GLMs) to examine how fire and vegetation type affect termite species at the landscape scale (predictor variables outlined in table 1). The responses of individual species were modelled in two ways, depending on the species' prevalence. First, for species that occurred in greater than 20% of study landscapes, and presented a range of values in terms of the number of sites occupied, we modelled the proportion of sites each species occurred at within a landscape (i.e. number of sites occupied/total number of sites; Nimmo *et al*. [18]). For species that occurred in greater than 20% of landscapes, but which occurred in few sites within most landscapes, we modelled their landscape-scale presence/absence (due to there being too little variation in the proportion of sites occupied within landscapes). Both response variables were assumed to follow a binomial distribution [44]. Species richness was assumed to follow a Gaussian distribution because it provided a better fit to the data at this scale (more normally distributed residuals) compared with a Poisson distribution.

At the landscape scale, fire history was represented by the proportional extent of post-fire-age classes (following [12]) (table 1). ‘Fire diversity’ (i.e. pyrodiversity) was calculated using the Shannon–Weiner diversity index based on the proportion of fire-age classes present in each landscape [12]. Examination of pairwise collinearity between predictor variables revealed that the extent of the mid-successional fire-age-class was significantly correlated (greater than 0.6) with that of recently burned, and hence the former was excluded from analysis. The proportional cover of heathy mallee and dunefield heath were highly negatively correlated (*r*_p _= −0.84). Therefore, only one of these two measures was included in a single model, based on QAIC_c_ (i.e. the predictor that produced the lowest QAIC_c_ was included in subsequent models). Predictor variables were log-transformed to consider nonlinear relationships. The fit of the linear and log-transformed models was compared to assess the best fit for each response variable using QAIC_c_ [47].

As with the site-level GLMMs, model selection was undertaken using an information theoretic approach [47]. Continuous predictor variables were again standardized (mean = 0, s.d. = 1) to allow direct comparison of coefficient estimates, and variables for which 85% confidence intervals did not cross zero were regarded as important [49]. All analyses were run in R v. 3.1.0 using the ‘mgcv’, ‘lme4’ and ‘MuMin’ packages [51–53].

## 3. Results

Termites from nine species were recorded in total. Termite activity was recorded at 91 of the 100 sites based on both active searches and baits (electronic supplementary material, table S1). Species richness at the site scale ranged from 1 to 5 species, with an average of 1.75 species. At the landscape scale, species richness ranged from 2 to 8, with an average of 3.81 species. Species detection varied between baits and active searches, with several species only detected during active searches, and only one species commonly encountered on baits (electronic supplementary material, table S1). Termite attack on cellulose baits was recorded at 82 of the 100 sites on 154 of the 600 rolls (approx. 25%). One species (*Heterotermes ferox*) made up the bulk of identifiable observations (soldier castes) from the baits (electronic supplementary material, table S1). One other species (*Coptotermes frenchi*) was located on baits at one site only (electronic supplementary material, table S1). No other species were recorded from baits. The active search survey method detected a greater number of species with a total of nine species being located across the study area (electronic supplementary material, table S1). Five individual species were recorded frequently enough for analysis: *Heterotermes ferox*, *Nasutitermes exitiosus*, *Microcerotermes* sp. 1, *Coptotermes frenchi* and *Amitermes* sp.

### 3.1. Does fire affect termite habitat resources?

**Figure 3.**
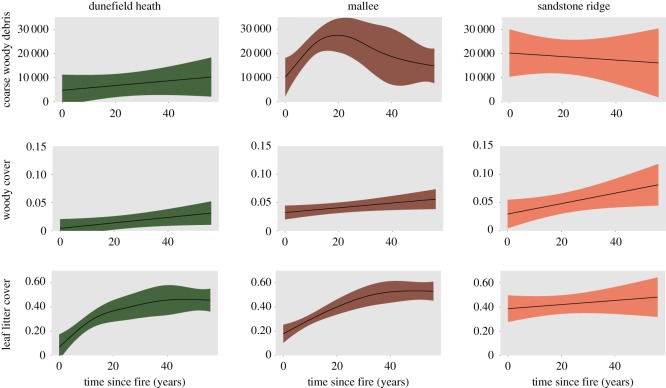
The responses of habitat resources to time-since-fire across a 56-year chronosequence within the three dominant vegetation communities in the Big Desert study region. The predicted response curves are represented by the black lines, and the 95% confidence intervals are represented by green for dunefield heath, brown for mallee and orange for sandstone ridge shrubland. Coarse woody debris is measured as volume (cubic centimetres); while woody vegetation cover and leaf litter cover are proportional cover values. Only habitat resources with a significant relationship with either time-since-fire or vegetation type are shown.

Three of the five habitat variables showed a significant response to time-since-fire in at least one vegetation type (table 2). The volume of coarse woody debris showed a significant response to time-since-fire, but only within heathy mallee vegetation, where it increased until approximately 20 years post-fire (figure 3). Differences between vegetation types were also evident, as heathy mallee and sandstone ridge shrubland had higher volumes of woody debris compared to dunefield heath, which was the reference category throughout (table 2). Woody vegetation cover showed a significant and positive response to time-since-fire in both heathy mallee and sandstone ridge shrubland vegetation (table 2). Differences were again evident between vegetation types, with higher woody vegetation cover in heathy mallee and sandstone ridge compared to dunefield heath (table 2). Leaf litter cover showed a significant positive response to time-since-fire in dunefield heath and heathy mallee vegetation (table 2). Among vegetation types, leaf litter cover was higher overall in sandstone ridge shrubland (table 2). Habitat complexity and Triodia did not show any response to time-since-fire (table 2) or vegetation type.

**Table 2.** Results of GAMMs describing the relationship between time-since-fire and termite habitat resources within each of the vegetation communities. Smoothed terms show the effects of time-since-fire on habitat attributes within each of the three vegetation types, while the linear predictor shows differences in habitat attributes between vegetation types. Significant results are shown in italics. Dunefield heath was specified as the reference category for vegetation type.

		significance of smoothed terms			significance of linear predictor				
response variable	vegetation community	edf^a^	*F*	*p-*value	vegetation type	coef	s.e.	*z*-value	*p-*value
Triodia cover	dunefield heath	1.000	0.843	0.361	intercept (dunefield heath)	0.011	0.006	1.848	0.068
	mallee	1.000	1.078	0.302	mallee	0.001	0.007	0.201	0.841
	sandstone ridge	1.000	0.002	0.967	sandstone ridge	−0.012	0.011	−1.083	0.282
coarse woody debris	dunefield heath	1.000	0.969	0.327	intercept (dunefield heath)	6696.000	2449.000	2.735	0.007
	*mallee*	*2*.*660*	*6*.*385*	*0*.*001*	*mallee*	*13300*.*000*	*2586*.*000*	*5*.*144*	*0*.*000*
	sandstone ridge	1.000	0.173	0.678	*sandstone ridge*	*12099*.*000*	*4086*.*000*	*2*.*961*	*0*.*004*
woody cover	dunefield heath	1.000	3.548	0.063	intercept (dunefield heath)	0.014	0.006	2.272	0.025
	*mallee*	*1*.*000*	*4*.*165*	*0*.*044*	*mallee*	*0*.*027*	*0*.*007*	*4*.*040*	*0*.*000*
	*sandstone ridge*	*1*.*000*	*4*.*165*	*0*.*044*	*sandstone ridge*	*0*.*034*	*0*.*011*	*3*.*239*	*0*.*002*
habitat complexity	dunefield heath	1.000	3.242	0.075	intercept (dunefield heath)	0.036	0.003	11.391	0.000
	mallee	1.000	3.298	0.073	mallee	0.000	0.003	0.002	0.998
	sandstone ridge	1.000	1.316	0.254	sandstone ridge	−0.002	0.004	−0.572	0.569
leaf litter	*dunefield heath*	*2*.*348*	*14*.*314*	*0*.*000*	intercept (dunefield heath)	0.292	0.025	11.715	0.000
	*mallee*	*1*.*972*	*21*.*247*	*0*.*000*	mallee	0.054	0.029	1.855	0.067
	sandstone ridge	1.000	0.704	0.404	*sandstone ridge*	*0*.*127*	*0*.*046*	*2*.*780*	*0*.*007*

^a^Estimated degrees of freedom.

### 3.2. Does vegetation structure influence termite occurrence?

**Figure 4.**
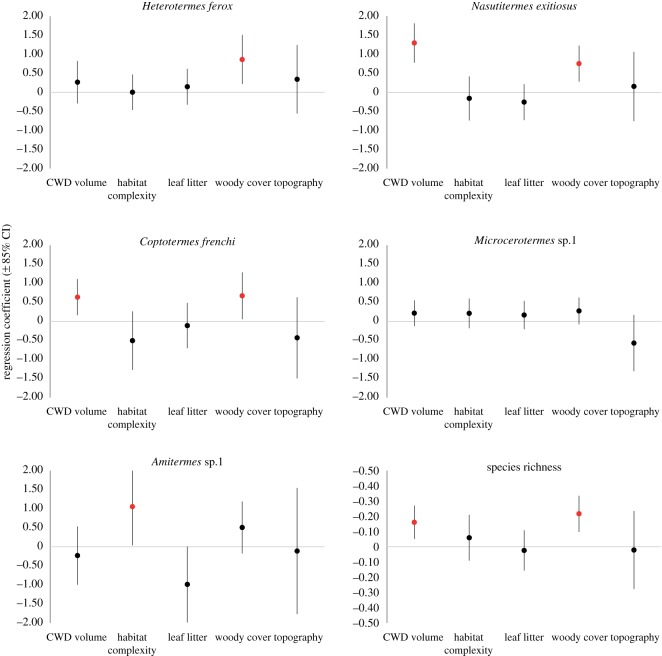
Regression coefficients, indicated in black circles, and associated 85% confidence intervals for GLMMs of termite species occurrence and species richness. Associated habitat predictor variables are considered important if the 85% confidence interval does not overlap zero (red circles). Confidence intervals above the zero line indicate a positive influence, while those below the zero line indicate a negative influence.

No clearly best model was identified for any of the individual species or species richness, and so model averaging was conducted. Coefficient estimates from model averaging indicated that woody vegetation cover had a positive coefficient with 85% confidence intervals not overlapping zero for *H. ferox*, *C. frenchi* and *N. exitiosus* (figure 4). The volume of coarse woody debris was also identified as important for *C. frenchi* and *N. exitiosus* (i.e. 85% confidence intervals did not overlap zero) (figure 4). All habitat variables had 85% confidence intervals that overlapped zero for *Microcerotermes* sp. 1. Coarse woody debris and woody vegetation both had a positive influence on species richness and 85% confidence intervals that did not overlap zero (figure 4).

### 3.3. Does fire affect termite occurrence?

GAMMs indicated fire was not significantly related to the occurrence of any individual termite species or species richness in any vegetation type (table 3). Vegetation type had a significant influence on three species (*H. ferox, N. exitiosus* and *Microcerotermes* sp. 1), all of which were more likely to occur in heathy mallee vegetation compared to dunefield heath, and *N. exitiosus* was also more likely to occur in sandstone ridge vegetation compared to dunefield heath (table 3). Species richness was also significantly higher in heathy mallee and sandstone ridge compared to dunefield heath vegetation (table 3).

**Table 3.** Results of GAMMs describing the relationship between time-since-fire/vegetation type and termite species occurrence/species richness. Smoothed terms show the effects of time-since-fire on species’ occurrence and richness within each of the three vegetation types, while the linear predictor shows differences in species’ occurrence and richness between vegetation types. Significant effects are shown in italics. Dunefield heath was specified as the reference category for vegetation type.

		significance of smoothed terms			significance of linear predictor				
response variable	vegetation community	edf^a^	*F*	*p-*value	vegetation type	coef	s.e.	*z*-value	*p-*value
*Heterotermes ferox*	dunefield heath	1	0.439	0.508	intercept (dunefield heath)	0.7269	0.387	1.878	0.06
	mallee	1	0.028	0.867	*mallee*	*1*.*8737*	*0*.*6469*	*2*.*896*	*0*.*004*
	sandstone ridge	1	1.444	0.23	sandstone ridge	2.9883	2.8638	1.043	0.297
*Amitermes* sp. 1	dunefield heath	1	0.121	0.728	intercept (dunefield heath)	−4.096	1.633	−2.508	0.012
	mallee	1	0.863	0.353	mallee	0.875	1.871	0.468	0.64
	sandstone ridge	1	0.403	0.526	sandstone ridge	1.796	2.045	0.878	0.38
*Microcerotermes* sp.1	dunefield heath	1	0.639	0.424	intercept (dunefield heath)	1.087	1.087	−2.772	0.006
	mallee	1	0.027	0.869	*mallee*	*1*.*121*	*1*.*121*	*2*.*186*	*0*.*029*
	sandstone ridge	1	0.458	0.498	sandstone ridge	1.293	1.293	1.543	0.123
*Nasutitermes exitiosus*	dunefield heath	1	0.245	0.621	intercept (dunefield heath)	−3.847	1.447	−2.659	0.008
	mallee	1	1.302	0.254	*mallee*	*3*.*714*	*1*.*467*	*2*.*532*	*0*.*011*
	sandstone ridge	1	0.437	0.509	*sandstone ridge*	*4*.*049*	*1*.*601*	*2*.*53*	*0*.*011*
*Coptotermes frenchi*	dunefield heath	1	0.000	1.000	intercept (dunefield heath)	−20.56	5232.47	−0.004	0.997
	mallee	1	0.048	0.826	mallee	18.86	5232.47	0.004	0.997
species richness	dunefield heath	1	0.109	0.741	intercept (dunefield heath)	−0.1353	0.1984	−0.682	0.495
	mallee	1	0.236	0.627	*mallee*	*0*.*9011*	*0*.*2171*	*4*.*151*	*0*.*000*
	sandstone ridge	1	0.021	0.884	*sandstone ridge*	*0*.*7588*	*0*.*2993*	*2*.*535*	*0*.*011*

^a^Estimated degrees of freedom.

### 3.4. Does the diversity of fire history within a landscape positively affect termite species richness?

**Figure 5.**
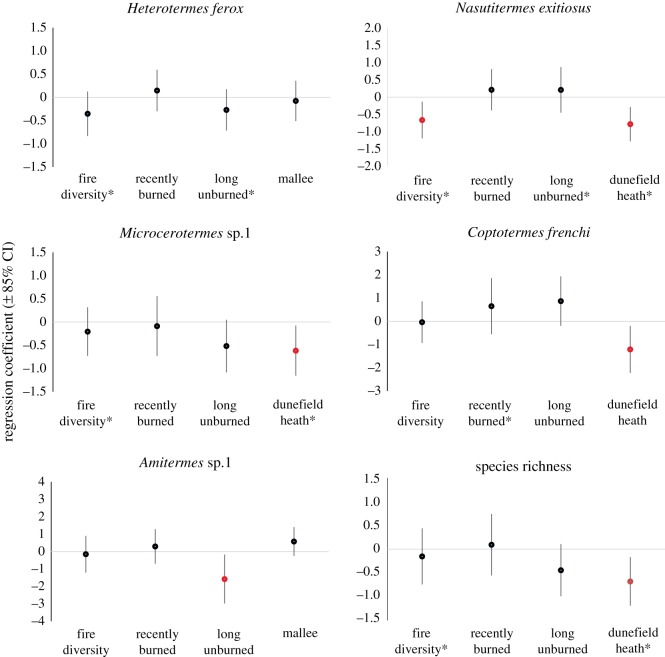
Regression coefficients, indicated in black circles, and associated 85% confidence intervals for GLMs of termite species occurrence and species richness. Associated habitat predictor variables are considered important if the 85% confidence interval does not overlap zero (red circles). Non-overlapping confidence intervals above the zero line indicate a positive influence, while those below the zero line indicate a negative influence. An asterisk indicates the variable was log-transformed.

**Figure 6.**
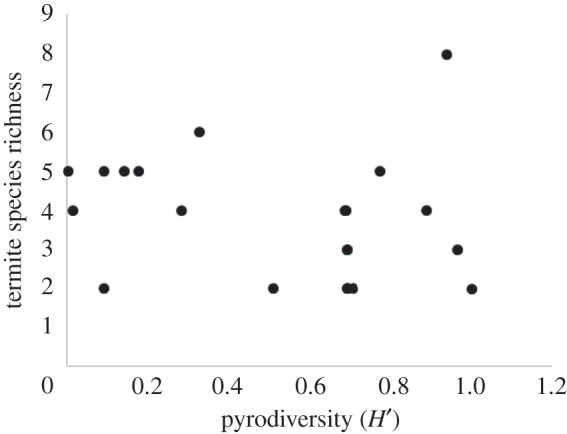
The relationship between termite richness and pyrodiversity (Shannon's diversity of fire-age classes) at the landscape scale in semi-arid southern Australia.

As with the site level analysis, no model was identified as clearly best for any individual termite species or termite species richness. Model averaging revealed that no landscape variables strongly influenced *Heterotermes ferox*, as the model-averaged coefficients all had 85% confidence intervals that overlapped zero (figure 5). Model averaging showed *N. exitiosus* had a negative relationship with both the extent of dunefield heath vegetation and the diversity of fire-ages within a landscape (figure 5). The extent of dunefield heath also negatively influenced *Microcerotermes* sp. 1 and *Coptotermes frenchi*. The only species affected by the extent of a fire-age class was *Amitermes* sp. 1, which was negatively related to the extent of long unburned vegetation (figure 5). Species richness was negatively related to the extent of dunefield heath vegetation (figure 5), and was not related to any fire-related properties of the landscapes, including pyrodiversity (figure 6).

## 4. Discussion

### 4.1. Does fire affect termite habitat resources?

We found that fire strongly affects a range of resources assumed *a priori* to be important to termites, including the volume of coarse woody debris, woody plant cover and leaf litter cover. All of these resources were less abundant immediately post-fire and accumulated thereafter, and in most cases changes continued throughout the 56 year post-fire chronosequence. This reiterates findings from studies to the north of our study region (the Mallee Fire and Biodiversity Project [54]), which showed that many habitat attributes continue to change for over a century following fire [19,55]. Also consistent with those and other studies [56–58] was the finding that the response of habitat attributes to fire is context dependent—in this instance, different rates of recovery of habitat attributes were apparent in different vegetation types. Despite differences between vegetation types, the strong influence of fire on several habitat attributes and the longevity of these effects mean that, in our study region, assumption 1 of the pyrodiversity hypothesis is supported.

### 4.2. Does vegetation structure influence termite occurrence?

Assumption 2 of the pyrodiversity hypothesis is that species within the taxonomic group of interest—in this case termites—are strongly linked to habitat attributes that are themselves shaped by fire. Four of the five species we studied were significantly related to at least one habitat attribute, with the volume of coarse woody debris and woody plant cover being most important. These two habitat attributes were themselves influenced by fire, thus providing some support for assumption 2 of the pyrodiversity hypothesis.

Our findings are similar to those from semi-arid southwestern Australia, where the number of trees and amount of woody debris were identified as the most important factors explaining termite species richness [41], and from a study to the north of our study region which found large pieces of dead wood (greater than 6 cm diameter) hosted more termite species and that several individual species were positively associated with the density of logs [29]. Termites in our study region rarely build mound structures; instead, this community is composed mostly of subterranean species that build nest structures and create tunnels underground. Subterranean termites are reliant on woody debris not only for food [28], but to also provide adequate shelter from temperature extremes and predators while they feed [59]. Woody debris is potentially a less important resource for mound-building termite species, which feed on humus in the safety of the mound structure [60], and for grass-harvesting, mound-building termites [61].

### 4.3. Does fire affect termite occurrence?

As the occurrence of four species was influenced by resources that also constitute fuel sources affected by fire, it was expected that the occurrence of these species would also be influenced by fire. However, none of the five species nor species richness was related to fire history, therefore violating assumption 3 of the pyrodiversity hypothesis. How can we explain this apparent discrepancy? As shown in this study, fire in mallee does not completely consume woody debris—even recently burned heathy mallee vegetation contains large volumes of woody debris relative to other vegetation types within the region, generally in the form of charred logs. Termites have been shown to survive feeding on charred wood [62], and there is also likely to remain abundant below-ground woody resources, particularly in sites with mallee trees due to their large underground lignotubers that are buffered from the effects of fire. Thus, even recently burned heathy mallee sites may have sufficient food to allow these species to persist.

In contrast to fire history, vegetation type did affect the occurrence of three termite species and species richness, all of which were less common in dunefield heath. Dunefield heath had the lowest volumes of woody debris and lower woody cover compared to heathy mallee sites. In fact, recently burned heathy mallee sites have volumes of woody debris and woody cover roughly equivalent to long unburned dunefield heath sites (figure 3). It is plausible then that the amount of woody resources in dunefield heath sites typically falls below the threshold required for those species to persist, and heathy mallee sites—regardless of age—typically fall above this threshold. Alternatively, the absence of several species from dunefield heath might reflect the kinds of woody resources available, due to compositional differences in the plant species that comprise that vegetation type (i.e. small shrubs as opposed to mallee trees).

Several studies also have found that termites are resistant towards the effects of fire [29,40,63,64]. However, some studies in both arid and tropical savannahs have observed negative responses, such as lower abundances following fire [24,65,66]. Studies reporting lower abundances of termites following fire often focused on mound-building termites, comprising harvester and fungus-growing species [24,65,66]. While direct mortality of individuals feeding in woody debris may occur [40], underground colonies of subterranean termites might be more buffered from the immediate influence of fire. Fire produces stark temperature gradients in mallee soils [67]—for example, decreasing from 100°C at 0.5 cm deep, to 30°C at only 4 cm deep [68]. Reported lethal temperature limits for some termite species indicate survival occurs at 2 cm or deeper underground [62]. Owing to the subterranean nature of mallee termite nests, the majority of the colony is, therefore, likely to be buffered from fire events. Thus, the traits of mallee termites seem pivotal to their (lack of) response to fire.

### 4.4. Does the diversity of fire history within a landscape positively affect termite species richness?

A key assumption of the pyrodiversity hypothesis is that fire strongly affects the distribution of species, and that different species peak in their probability of occurrence or abundance at different stages following fire. However, termite species in this study were not found to be associated with fire, and their probability of occurrence did not peak within any post-fire stages. It is, therefore, unsurprising that the properties of fire mosaics—including the diversity of fire-age classes—were not important factors affecting termite occurrence or richness, thus rejecting the pyrodiversity hypothesis.

#### 4.4.1. The pyrodiversity hypothesis

By elucidating and testing several of the assumptions underlying the pyrodiversity hypothesis, we have been able to reveal the point at which this hypothesis breaks down for termites in our region. In our case, two of the critical assumptions were met—habitat resources that are shaped by fire (assumption 1) impacted on the distribution of several species (assumption 2)—but this did not translate into those same species having distributions that are tightly linked to fire history. While we have offered an ecological explanation for why this occurred, it is important to note that this break down may be partly due to our measure of termite occurrence being presence/absence. Had we modelled the abundance of termites as opposed to presence/absence, we may have come to different conclusions. However, measuring the abundance of termites is notoriously difficult due to their subterranean lifestyle, locally patchy distributions and the impracticality of conventional methods of abundance estimation (e.g. mark–recapture).

Our finding that termites are linked to resources affected by fire suggests caution is required when interpreting our results in terms of fire management. Although we found no evidence that time-since-fire affects termite occurrence, it is possible—perhaps likely—that other aspects of the fire regime may well impact termite distributions through their impacts on habitat resources [55,69]. For example, high severity fires occurring during drier periods might incinerate more woody resources than low severity fires occurring during wetter periods [70]. Similarly, short intervals between fires may diminish woody resources, affecting termite distributions in turn. For instance, Bassett *et al*. [56] showed that intense and frequent fire reduced the volume of coarse woody debris in foothill forests of southern Australia. Thus, there is potential for interactions between aspects of the fire regime to affect termite distributions, even though time-since-fire alone does not.

For other taxa within and beyond our region, a lack of support for the pyrodiversity hypothesis may arise through the violation of other assumptions. For example, assumption 2 might be violated in highly fire-prone ecosystems where biota have adapted a high degree of resistance to frequent fires [4], including having less reliance on resources that are consumed by fire [14,30]. On the other hand, other studies have failed to see an effect of pyrodiversity on species diversity even when assumptions 1–3 have been met [13,50,71,72]. In those instances, it was not pyrodiversity *per se* that would promote species diversity, but a specific mix of fire-ages linked to key resources [73]. There are further assumptions of the pyrodiversity hypothesis that we did not explicitly assess, such as the impact of the spatial scale of pyrodiversity—Bird *et al*. [74] showed that areas under indigenous fire regimes had a similar mix of fire-ages compared to those under a lighting regime, but the spatial scale at which fire histories co-occurred was much finer under the indigenous fire regime. The scale of fire mosaics could influence the ability of species to colonize suitable fire-ages and establish populations, or, for more mobile species, to reach multiple fire-ages on a daily basis and thus access multiple resources. Thus, the scale at which pyrodiversity is generated (and measured) is a further factor that can influence whether or not pyrodiversity begets biodiversity, and the scale at which such relationships can be observed.

On close inspection, the intuitive hypothesis that ‘pyrodiversity begets biodiversity’ is laden with assumptions that need to be met before a clear relationship can be seen. This might explain why studies to date have seen such mixed results, and why many studies have failed to see patterns consistent with the hypothesis [12–15].

#### 4.4.2. Management implications

Our results do not support patch-mosaic burning as a management strategy for enhancing termite species richness. The study species are largely resistant to the effects of time-since-fire, thus management actions that create a variety of fire-age patches are unlikely to impact their occurrence, either positively or negatively. Despite time-since-fire not being a key driver of termite occurrence or richness in the Big Desert, fire management must meet the needs of all species, not just termites. There is little understanding of the potential impacts of frequent burning on other taxonomic groups within the Big Desert, particularly vertebrate fauna. Research from mallee ecosystems north of the Big Desert suggests that many species rely on long-unburned habitat resources [18,50,75]. This is particularly the case for birds and some reptiles that rely on resources associated with older successional stages, such as hollows, leaf litter and decorticating bark for foraging or shelter [72,75]. As many species that are negatively influenced by fire to the north of the study region also occur within the Big Desert, it is possible that increasing the frequency of fire in the Big Desert may have negative effects on these late-succession-dependent fauna. However, more research on this region's biota is required to allow for evidence-based fire management for biodiversity conservation.

## Supplementary Material

Comparison of detection methods

## Data accessibility

Raw data are accessible through Dryad Digital Repository (http://dx.doi.org/10.5061/dryad.mh084f6) [76].
