# Supplementary material for: Testing the assumptions of the pyrodiversity begets biodiversity hypothesis for termites in semi-arid Australia
Source: R Soc Open Sci. 2018 Apr 25;5(4):172055. doi: 10.1098/rsos.172055 (PMC5936926; doi:10.1098/rsos.172055)
Supplement: Comparison of detection methods [file rsos172055supp1.docx]

**Table S1.** Summary of termite species located in the Big Desert study region, including the number of sites species were located on toilet paper roll baits and during active search surveys, as well as the total number (including both survey methods) of sites and landscapes each species was located at.

| **Termite species** | **Baits (/100)** | **Active search (/100)** | **Sites**  **Located (/100)** | **Landscapes located**  **(/20)** |
| --- | --- | --- | --- | --- |
| *Heterotermes ferox* | 59 sites | 68 sites | 83 sites | 20 landscapes |
| *Nasutitermes exitiosus* | 0 sites | 35 sites | 35 sites | 16 landscapes |
| *Microcerotermes* sp. 1 | 0 sites | 26 sites | 26 sites | 15 landscapes |
| *Coptotermes frenchi* | 1 site | 12 sites | 12 sites | 9 landscapes |
| *Amitermes* sp. 1 | 0 sites | 9 sites | 9 sites | 6 landscapes |
| *Microcerotermes cavus* | 0 sites | 5 sites | 5 sites | 4 landscapes |
| *Kalotermes* sp. 1 | 0 sites | 3 sites | 3 sites | 3 landscapes |
| *Amitermes modicus* | 0 sites | 1 site | 1 site | 1 landscape |
| *Ephelotermes argutus* | 0 sites | 1 site | 1 site | 1 landscape |
